# The Use of Outpatient Diuretics Does Not Impact Somatic Growth in Infants With Bronchopulmonary Dysplasia

**DOI:** 10.1002/ppul.71712

**Published:** 2026-06-23

**Authors:** Brianna C. Aoyama, Sharon A. McGrath‐Morrow, Joseph M. Collaco

**Affiliations:** ^1^ Eudowood Division of Pediatric Respiratory Sciences Johns Hopkins University School of Medicine Baltimore Maryland USA; ^2^ Division of Pulmonary Medicine Children's Hospital of Philadelphia and University of Pennsylvania Perelman School of Medicine Philadelphia Pennsylvania USA

To the Editor,

Bronchopulmonary dysplasia (BPD) continues to be one of the most common complications of preterm birth. Despite advances in neonatal care and technology, infants with BPD exhibit ongoing inflammation, impaired alveolarization, increased airway resistance, and pulmonary edema, all of which contribute to increased work of breathing and higher metabolic demand. Diuretics are widely used in neonatal intensive care units (NICUs) to decrease pulmonary interstitial edema, enhance lung compliance, improve oxygenation, and lower metabolic demands, potentially supporting somatic growth in infants whose caloric expenditure is dominated by respiratory effort. Loop diuretics have been shown to transiently reduce airway resistance and enhance lung compliance, whereas thiazide diuretics and potassium‐sparing agents are commonly used for longer‐term outpatient management due to their more favorable safety. Despite the potential physiologic benefits, evidence supporting the routine or prolonged use of diuretics in BPD and their effect on somatic growth remains inconsistent [1, 2], and practice patterns vary significantly among institutions [3]. Chronic diuretic exposure is associated with electrolyte disturbances, impaired appetite, and disrupted protein accretion. Loop diuretics specifically increase urinary calcium losses and have been linked to nephrocalcinosis, osteopenia of prematurity, and impaired linear growth. Diuretic‐induced fluid shifts may further complicate nutritional management in infants already struggling with feeding intolerance. Thus, while diuretics may provide short‐term respiratory improvement that facilitates growth, their long‐term use has the potential to hinder somatic growth. This study aims to assess this balance between physiologic benefit and metabolic cost in infants with BPD treated with outpatient diuretics, with the goal of optimizing therapeutic strategies for infants with BPD.

## 1 Methods

### 1.1 Study Population

Subjects for this retrospective cohort study were recruited from outpatient BPD clinics at the Children's Hospital of Philadelphia and Johns Hopkins Children's Center between 2008 and 2023. Inclusion criteria included being born at or less than 32 weeks of gestation, a diagnosis of BPD by the 2001 National Institutes of Health (NIH) consensus definition, and recorded weights prior to 3 years of age. Exclusion criteria included a diagnosis of cyanotic congenital heart disease. This study was approved by the Children's Hospital of Philadelphia Institutional Review Board (IRB # 20‐017614_AM18) and Johns Hopkins University IRB (Protocol #NA_00051884). All caregivers provided informed consent in accordance with IRB‐approved protocols.

### 1.2 Data Collection

Data were obtained through chart review. Subjects had at least one recorded weight within 6 months before and after diuretic discontinuation as an outpatient. In the case of control subjects who were not prescribed diuretics after NICU discharge, weights were collected for 6 months preceding and following 9.23 months chronological age, representing the median age of diuretic discontinuation in the treatment group. Weights were converted to CDC *z*‐scores and averaged for the relevant periods. Birthweight percentile was corrected by gestational age. Pulmonary hypertension was defined as present on echocardiogram or cardiac catheterization on or after 36 weeks corrected age. Median household income was estimated from residential zip codes using the United States Census data.

### 1.3 Statistical Analysis

Chi‐square tests and *t‐*tests were used to compare demographic and clinical characteristics between subjects on and not on outpatient diuretic therapy. Weight *z*‐scores were compared similarly. Clustered (by site) unadjusted and adjusted regressions were performed to assess differences between mean weight *z*‐scores between those on and not on outpatient diuretic therapy. Adjusted regressions were adjusted for potential confounders selected a priori, including gestational age, birthweight, BPD severity, pulmonary hypertension, and gastrostomy tube. Results with a *p* value less than or equal to 0.05 were considered statistically significant. Stata 15.0 was used for data analysis (StataCorp, College Station, TX).

## 2 Results

### 2.1 Demographics

Data were collected from 829 children, 459 (55.4%) of whom were prescribed diuretics at their first outpatient visit. Baseline demographic and clinical characteristics are summarized in Table 1. Forty‐five percent of the study population was female, 61.3% identified as non‐White, and 8.2% were of Hispanic ethnicity. Mean gestational age was 26.5 ± 2.2 weeks, mean age at NICU discharge was 5.0 ± 2.8 months, and mean age at first pulmonary visit was 7.3 ± 3.7 months. Children prescribed diuretic therapy in the outpatient setting had lower birth weights (832 vs. 877 g, *p* = 0.031), were older at the time of initial discharge (5.4 vs. 4.5 months, *p* < 0.001), and had more severe BPD (69.9% vs. 51.4%, *p* < 0.001) than children not on diuretic therapy. Markers of more significant lung disease (need for tracheostomy, home ventilator, home supplemental oxygen) and associated comorbidities (pulmonary hypertension, dysphagia represented as need for gastrostomy and Nissen fundoplication) were more prevalent in the diuretic group.

**Table 1:** Study population demographics.

Mean ± SD [range]		Study population (*n* = 829)	Subjects not on outpatient diuretics (*n* = 370)	Subjects on outpatient diuretics (*n* = 459)	*p* value
Sex (% female)		45.0%	43.3%	45.5%	0.73
Race (% yes; *n* = 827)	Asian	6.1%	6.8%	5.5%	0.42
	Black	49.8%	49.2%	50.3%	0.74
	Native American	0.2%	0.0%	0.4%	0.21
	White	38.7%	41.3%	36.6%	0.17
	Other	7.7%	7.3%	8.1%	0.70
Ethnicity (% Hispanic; *n* = 825)		8.2%	10.4%	6.5%	**0.046**
Median household income ($'000s)		71.9 ± 28.8	72.2 ± 30.2	71.7 ± 27.6	0.77
		[13.8, 213.7]	[16.1, 213.7]	[13.8, 156.8]	
Public insurance (% yes)		55.1%	55.7%	54.7%	0.78
Gestational age (weeks)		26.5 ± 2.2	26.6 ± 2.2	26.4 ± 2.2	0.18
		[22.3, 32.0]	[22.3, 32.0]	[22.4, 32.0]	
Birth weight (g; *n* = 828)		852 ± 296	877 ± 309	832 ± 284	**0.031**
		[380, 2100]	[390, 2100]	[380, 1900]	
Birth weight percentile (%; *n* = 828)		42 ± 26	43 ± 26	42 ± 26	0.57
		[1, 95]	[1, 94]	[1, 95]	
BPD severity (% yes)	Mild	11.0%	14.9%	7.8%	**< 0.001**
	Moderate	25.9%	31.1%	21.8%	
	Severe	61.6%	51.4%	69.9%	
	Unknown	1.5%	2.7%	0.4%	
Age at initial hospital discharge (months)		5.0 ± 2.8	4.5 ± 2.5	5.4 ± 2.9	**< 0.001**
		[0.9, 19.5]	[1.1, 15.8]	[0.9, 19.5]	
Age at first pulmonary visit (months)		7.3 ± 3.7	7.4 ± 4.3	7.2 ± 3.2	0.29
		[1.3, 32.6]	[1.3, 32.6]	[1.4, 21.1]	
Tracheostomy (% yes)		8.8%	6.0%	11.1%	**0.009**
Home ventilator (% yes)		8.3%	5.4%	10.7%	**0.006**
Home oxygen (% yes)		43.2%	39.2%	46.4%	**0.037**
Gastrostomy (% yes)		34.7%	26.5%	41.4%	**< 0.001**
Nissen fundoplication (% yes)		17.5%	11.4%	22.4%	**< 0.001**
Pulmonary hypertension (% yes)		21.0%	15.7%	25.3%	**0.001**
Outpatient pulmonary hypertension medications (% yes)		8.3%	4.6%	11.3%	**< 0.001**
Outpatient inhaled steroids (% yes)		62.5%	52.2%	70.8%	**< 0.001**

*Note:* Values in bold denote statistically significant differences (*p* < 0.05).

### 2.2 Somatic Growth

Mean weight *z*‐scores before and after diuretic discontinuation were compared between those children prescribed outpatient diuretics and those not on chronic diuretic therapy (Table 1). Children on diuretic therapy had lower mean weight *z*‐scores before discontinuation (−2.62 vs. −2.87, *p* = 0.008), but this difference did not persist after discontinuation (−1.98 vs. −1.9, *p* = 0.37); however, children not prescribed diuretics demonstrated a statistically significant improvement in weight *z*‐score after diuretic discontinuation (or similar timepoint) compared to children on diuretic therapy (0.97 vs. 0.64, *p* < 0.001). Comparing children on one versus two or more diuretics (Supporting Information S1: Table 1), there was no significant difference in mean weight *z*‐score before or after diuretic discontinuation; however, children on one diuretic had significantly more weight measurements before and after diuretic discontinuation compared to children on two or more diuretics. In unadjusted site‐clustered analysis, there was no difference in weight *z*‐score before or after diuretic discontinuation (or similar timepoints) or improvement in *z*‐scores before and after diuretic discontinuation (or similar timepoints) between groups (Table 2). After adjusting for gestational age, birth weight, BPD severity, pulmonary hypertension, and gastrostomy tube, differences remained nonsignificant. In a sensitivity analysis restricted to children with grade 3 BPD (Supporting Information S1: Tables 2 and 3), results were consistent with the primary analysis.

**Table 2:** Difference in *Z*‐scores with discontinuation of diuretics compared to similar timepoints in the control (no diuretic) group^b^ .

Coefficient ± SE [95% CI]	Unadjusted regression^a^ (*n* = 829)	*p* value	Adjusted regression^b^ (*n* = 828)	*p* value
Difference in weight *z*‐score from baseline to timepoint 1 between no diuretic versus diuretic^c^	−0.25 ± 0.18 [−2.08, 2.59]	0.40	0.16 ± 0.17 [−1.95, 2.26]	0.52
Difference in weight *z*‐score from timepoint 1 to timepoint 2 between no diuretic versus diuretic^c^	−0.09 ± 0.02 [−0.29, 0.11]	0.12	−0.09 ± 0.06 [−0.87, 0.68]	0.37
Difference in improvement in *z*‐scores between baseline and timepoint 2 between no diuretic versus diuretic^c^	−0.34 ± 0.17 [−2.47, 1.79]	0.29	−0.25 ± 0.23 [−3.13, 2.63]	0.47

^a^ All regressions were clustered by site. For all regressions, the independent variable was outpatient diuretic use, and the dependent variable was either the mean *z*‐score or the difference in *z*‐score.

^b^ Adjusted regressions were adjusted for gestational age, birth weight, BPD severity, presence of pulmonary hypertension on or after 36 weeks corrected age, and presence of gastrostomy tube a priori.

^c^ Weight *z*‐scores were calculated as the mean of all measurements obtained during the 6 months before and 6 months after diuretic discontinuation. For subjects not treated with diuretics, weight *z*‐scores were calculated during analogous 6‐month periods before and after 9.23 months chronological age, the median age of diuretic discontinuation in the diuretic‐treated group. Baseline = 6 months prior to diuretic discontinuation (or 3.23 months for those not on diuretics); timepoint 1 = time of diuretic discontinuation (or 9.23 months for those not on diuretics); timepoint 2 = 6 months after diuretic discontinuation (or 15.23 months for those not on diuretics).

## 3 Discussion

In this study, outpatient diuretic use was not associated with differences in somatic growth among infants and children with BPD, providing important reassurance regarding the growth safety profile of these commonly prescribed medications. At the same time, however, our findings do not necessarily support the continued use of diuretics to encourage growth and weight gain in this vulnerable population. The observed neutral growth effect may reflect a balance between the physiologic benefits of diuretics and their potential adverse consequences. By reducing pulmonary interstitial edema and improving lung compliance, diuretics may decrease the work of breathing and improve feeding tolerance, thereby facilitating adequate caloric intake and energy utilization that is especially important during a critical period of postnatal growth. At the same time, theoretical risks such as electrolyte imbalances, altered bone mineralization, and renal losses have raised concern for impaired weight gain. Our findings suggest that, in contemporary outpatient practice, these opposing influences may offset one another, resulting in preserved overall weight gain.

While previous inpatient‐based studies have raised concern about the use of diuretics slowing weight gain in infants and children with BPD [4], to our knowledge, this is one of the first studies to examine the association between diuretic use and somatic growth in the outpatient setting. Our findings are consistent with, and build upon, recent literature examining outpatient diuretic use in BPD [2, 5, 6] and support current international guidelines (American Thoracic Society 2021, European Respiratory Society 2020), which recommend against the routine use of diuretics and suggest natural weaning by allowing children to slowly outgrow their dose with increasing weight gain. Additionally, our findings suggest that appropriately managed outpatient diuretic therapy, including close surveillance of weight trends and frequent reevaluation of need to balance the respiratory advantages of diuretics while protecting somatic growth, may mitigate previously hypothesized risks to growth.

Ultimately, these findings reinforce that diuretics can remain a viable component of outpatient BPD management without imposing an additional burden on growth during a critical period of postnatal development. This has meaningful clinical implications, as maintaining adequate somatic growth is closely linked to improved neurodevelopmental outcomes and long‐term pulmonary function. Rather than necessitating routine discontinuation based on growth concerns alone, an individualized approach to diuretic management guided by respiratory status and clinical response remains a general best practice in this population. This study has several limitations. Its retrospective design and lack of granular data on diuretic dose, duration, and adherence limit our ability to detect cumulative effects on growth. Detailed nutritional data, including caloric density, fortification regimens, and use of supplements, were not systematically captured, and residual confounding by unmeasured nutritional interventions remains possible. Discharge weight *z*‐scores were unavailable, precluding analysis of growth trajectories during the period of active diuretic exposure between NICU discharge and diuretic discontinuation. Our cohort was drawn from two tertiary referral BPD centers enriched for severe disease and associated comorbidities, as reflected by the notably low pre‐discontinuation weight *z*‐scores, which may limit generalizability to populations with milder BPD and more favorable baseline growth. Additionally, the broad enrollment period (2008−2023) introduces the possibility of temporal confounding from evolving neonatal and outpatient management practices. Future prospective studies are needed to more definitively characterize the long‐term impact of chronic diuretic therapy on somatic growth, including the temporal relationship between diuretic exposure and growth, and should aim to identify specific subgroups that may experience optimal benefit from continued therapy, as well as define best practices for duration and monitoring in the outpatient setting.

## Author Contributions

**Brianna C. Aoyama:** conceptualization, writing – original draft, writing–review and editing, methodology, formal analysis, data curation, investigation. **Sharon A. McGrath‐Morrow:** conceptualization, investigation, writing – review and editing, formal analysis, methodology, data curation, funding acquisition. **Joseph M. Collaco:** conceptualization, investigation, writing – review and editing, data curation, funding acquisition.

## Conflicts of Interest

The authors declare no conflicts of interest.

## Supporting information

Supporting File

## Data Availability

The data that support the findings of this study are available on request from the corresponding author. The data are not publicly available due to privacy or ethical restrictions.
