# Supplementary material for: The Use of Outpatient Diuretics Does Not Impact Somatic Growth in Infants With Bronchopulmonary Dysplasia
Source: Pediatr Pulmonol. 2026 Jun 23;61(6):e71712. doi: 10.1002/ppul.71712 (PMC13288311; doi:10.1002/ppul.71712)
Supplement: Supplementary file 1 — Supporting File [file PPUL-61-0-s001.docx]

**Supplemental Table 1.** Weight Z Scores Before and After Diuretic Discontinuation in Subjects on 1 Diuretic versus Subjects on 2 or More Outpatient Diuretics

| Mean ± S.D.  [Range] | **Subjects on 1 Outpatient Diuretic**  (n = 250) | **Subjects on 2 or more Outpatient Diuretics**  (n = 209) | ***P* Value** |
| --- | --- | --- | --- |
| **Mean weight z score before diuretic discontinuation*** | -2.70 ± 1.35  [-6.87, 1.65] | -2.53 ± 1.43  [-5.54, 1.88] | 0.20 |
| **Number of weight measurements before diuretic discontinuation** | 13.4 ± 25.1  [1, 128] | 4.6 ± 7.4  [1, 93] | **<0.001** |
| **Mean weight z score after diuretic discontinuation*** | -2.03 ± 1.36  [-6.19, 2.82] | -1.94 ± 1.37  [-6.80, 3.45] | 0.48 |
| **Number of weight measurements after diuretic discontinuation*** | 5.5 ± 4.9  [1, 34] | 3.7 ± 2.8  [1, 30] | **<0.001** |
| **Improvement in z scores between before and after diuretic discontinuation*** | 0.67 ± 0.97  [-2.94, 4.15] | 0.59 ± 0.96  [-2.57, 4.11] | 0.41 |

*Weight z scores were averaged for the 6 months before and after the date of discontinuation of diuretics.

**Supplemental Table 2.** Weight Z Scores Before and After Diuretic Discontinuation (Severe BPD only)

| Mean ± S.D.  [Range] | **Subjects not on Outpatient Diuretics**  (n = 190) | **Subjects on Outpatient Diuretics**  (n = 321) | ***P* Value** |
| --- | --- | --- | --- |
| **Mean weight z score before diuretic discontinuation*** | -3.03 ± 1.33  [-7.98, 1.42] | -2.44 ± 1.40  [-6.87, 1.88] | **<0.001** |
| **Number of weight measurements before diuretic discontinuation** | 4.3 ± 1.7  [1, 9] | 10.9 ± 21.3  [1, 128] | **<0.001** |
| **Mean weight z score after diuretic discontinuation*** | -2.08 ± 1.47  [-6.16, 2.56] | -1.95 ± 1.34  [-6.80, 3.45] | 0.29 |
| **Number of weight measurements after diuretic discontinuation*** | 3.8 ± 1.7  [1, 6] | 5.1 ± 4.2  [1, 34] | **<0.001** |
| **Improvement in z scores between before and after diuretic discontinuation*** | 0.96 ± 1.08  [-2.30, 4.18] | 0.49 ± 0.89  [-2.57, 3.71] | **<0.001** |

*Weight z scores were averaged for the 6 months before and after the date of discontinuation of diuretics. For subjects not on diuretics, weights were averaged for 6 months before and after 9.23 months of chronological age; 9.23 months was the median age of diuretic discontinuation for subjects on diuretics.

**Supplemental Table 3.** Weight Z scores before and after Diuretic Discontinuation (Severe BPD only)

| Coefficient ± S.E.  [95% C.I.] | **Unadjusted Regression***  (n = 511) | ***P* Value** | **Adjusted Regression****  (n = 511) | ***P* Value** |
| --- | --- | --- | --- | --- |
| **Difference in weight z score before diuretic discontinuation between no diuretic vs. diuretic***** | 0.60 ± 0.04  [0.09, 1.11] | **0.043** | 0.47 ± 0.04  [0.004, 0.93] | 0.050 |
| **Difference in weight z score after diuretic discontinuation between no diuretic vs. diuretic***** | 0.13 ± 0.20  [-2.39, 2.66] | 0.62 | 0.07 ± 0.25  [-3.11, 3.25] | 0.82 |
| **Difference in Improvement in z scores between before and after diuretic discontinuation***** | -0.46 ± 0.24  [-3.50, 2.57] | 0.30 | -0.39 ± 0.29  [-4.03, 3.25] | 0.40 |

*All regressions were clustered by site. For all regressions, the independent variable was outpatient diuretic use and the dependent variable was either mean z score or difference in z score.

**Adjusted regressions were adjusted for gestational age, birth weight, presence of pulmonary hypertension on or after 36 weeks corrected age, and presence of gastrostomy tube *a priori*.

***Weight z scores were averaged for the 6 months before and after the date of discontinuation of diuretics. For subjects not on diuretics, weights were averaged for 6 months before and after 9.23 months of chronological age; 9.23 months was the median age of diuretic discontinuation for subjects on diuretics.
